# Cellular location and activity of *Escherichia coli* RecG proteins shed light on the function of its structurally unresolved C-terminus

**DOI:** 10.1093/nar/gku228

**Published:** 2014-04-01

**Authors:** Amy L. Upton, Jane I. Grove, Akeel A. Mahdi, Geoffrey S. Briggs, David S. Milner, Christian J. Rudolph, Robert G. Lloyd

**Affiliations:** 1 Centre for Genetics and Genomics, University of Nottingham, Queen's Medical Centre, Nottingham, NG7 2UH, UK; 2 Department of Biochemistry, University of Oxford, South Parks Road, Oxford OX1 3QU, UK; 3 School of Medicine, University of Nottingham, Queen's Medical Centre, Nottingham NG7 2UH, UK; 4 School of Health Sciences and Social Care, Division of Biosciences, Brunel University, Uxbridge, London UB8 3PH, UK

## Abstract

RecG is a DNA translocase encoded by most species of bacteria. The *Escherichia coli* protein targets branched DNA substrates and drives the unwinding and rewinding of DNA strands. Its ability to remodel replication forks and to genetically interact with PriA protein have led to the idea that it plays an important role in securing faithful genome duplication. Here we report that RecG co-localises with sites of DNA replication and identify conserved arginine and tryptophan residues near its C-terminus that are needed for this localisation. We establish that the extreme C-terminus, which is not resolved in the crystal structure, is vital for DNA unwinding but not for DNA binding. Substituting an alanine for a highly conserved tyrosine near the very end results in a substantial reduction in the ability to unwind replication fork and Holliday junction structures but has no effect on substrate affinity. Deleting or substituting the terminal alanine causes an even greater reduction in unwinding activity, which is somewhat surprising as this residue is not uniformly present in closely related RecG proteins. More significantly, the extreme C-terminal mutations have little effect on localisation. Mutations that do prevent localisation result in only a slight reduction in the capacity for DNA repair.

## INTRODUCTION

RecG is a double-stranded DNA translocase associated with the maintenance of genomic integrity in bacteria (1,2). It is present in almost all species examined (3,4). Early genetic studies suggested a role in promoting homologous recombination, a possibility consistent with the strong synergism between *recG* and *ruv* null alleles (1,5) and reinforced when the *Escherichia coli* RecG protein was shown to unwind Holliday junction structures (6,7). The mode of unwinding proved reminiscent of the branch migration activity displayed by the RuvAB component of the RuvABC Holliday junction resolvase complex (8–10). This has leant weight to the idea that RecG and RuvABC provide partially overlapping pathways for processing intermediates in homologous recombination (5).

However, subsequent studies showed that RecG targets a range of other substrates, including D-loops and R-loops, raising the possibility of alternative roles. It has a strong affinity for structures mimicking complete or partial replication forks (11–16), and can convert a fork to a Holliday junction (17–23). Coupled with evidence of a genetic interaction with PriA protein (24–28), these studies led to the proposal that RecG might catalyse the reversal or regression of stalled forks *in vivo*, enabling repair or bypass of the blocking lesion and the restart of replication, thus facilitating the completion of chromosome duplication (17). PriA initiates replisome assembly at branched DNA structures, thereby enabling replication to restart at sites remote from *oriC* (11,29). Fork regression has been investigated in detail in *E. coli* (30,31), and models of replication restart invoking such regression have become generally popular, with several eukaryotic helicases having been shown in recent years to have the potential to catalyse such a reaction *in vitro* (32–35). However, evidence for such pathways *in vivo* remains at best indirect (36). Furthermore, recent studies have indicated that much of the *recG* mutant phenotype is a pathological consequence of stable DNA replication (SDR), a form of unscheduled chromosome replication triggered by PriA-mediated replisome assembly (2,37–38). The initiation of SDR is particularly noticeable in the terminus area of the chromosome where forks normally meet to complete replication. It appears that fork collision may frequently generate 3′ flap structures that could be targeted by PriA (39). RecG has a high affinity for 3′ flaps and in conjunction with single-stranded DNA exonucleases may normally eliminate these structures, preventing their exploitation by PriA (37,39). As with fork regression, this role would place RecG at or near sites of DNA replication.

Here we present evidence consistent with the idea that *E. coli* RecG localises to sites of active replication and identify residues near the C-terminus of the protein that may facilitate this localisation. In addition, we demonstrate that the extreme C-terminus of RecG, which is not resolved in the crystal structure, is vital for DNA unwinding but not for DNA binding. Indeed, deleting or substituting the very last amino acid is sufficient to reduce unwinding activity to less than 5% of wild-type.

## MATERIALS AND METHODS

### Strains and plasmids

Bacterial strains are listed in Table 1. All constructs used for synthetic lethality assays are based on *E. coli* K-12 MG1655 *ΔlacIZYA* strains carrying derivatives of pRC7, an unstable *lac^+^*, mini-F plasmid that is easily lost (40). pJJ100 is a derivative of pRC7 encoding *recG^+^* (41), while pAM475 is a derivative encoding *polA^+^*. The wild-type *polA* coding sequence plus some 50 bp of upstream sequence was amplified by PCR from MG1655 (42) using primers incorporating flanking *Apa*I restriction sites and then inserted into pRC7 at the *Apa*I site within *lacI^q^*. pAM475 eliminates the sensitivity to UV light of a strain carrying a C-terminal deletion of *polA* (27), demonstrating that the cloned *polA^+^* gene is functional. pAM210 is a *recG^+^* derivative of the expression vector pT7-7 (43). pQW145 is a pAM210 derivative encoding RecG Q640R (20). To construct strains carrying mutant *recG* genes encoding RecG C-terminal deletions or substitutions, the relevant mutations were first generated by PCR amplification using primers carrying the required sequence alterations. The products were cloned into pQW145, replacing the 3′ end of *recG* with the new mutant sequence. The 3′ primer also added a *Hin*dIII site immediately downstream of the gene. A gene encoding resistance to kanamycin (*kan*) was then inserted at this site. The altered 3′ end of *recG* incorporating the desired deletion/substitution plus the downstream *kan* gene was amplified by PCR and the product used to engineer these features into strain MG1655, replacing the wild-type *recG* allele, using the protocol of Datsenko and Wanner (44). A strain encoding *recG^+^* linked to a downstream *kan* gene (*recG*^+^*-kan*) was engineered as a control for the C-terminal mutants generated. These *recG* alleles carrying the *kan* gene downstream are identified in Table 1 with the relevant alteration of the coding sequence shown in square brackets and with the suffix ‘*-kan*’. For ease of presentation, the suffix is omitted in the main text. For the same reason, the gene encoding RecG protein with both R682A and W683S substitutions is abbreviated to *recG[RW]*.

**Table 1.** *Escherichia coli* strains

Strain	Relevant genotype^a^	Source or reference^a^
STL5827	*F^−^ ompT hsdS* (*r_B_^−^ m_B_^−^*) *dcm gal λDE3*	Susan Lovett
AU1115	*ΔrecG::apra*	P1.N6052 × STL5827 to Apra^r^
AU1118	pLysS *ΔrecG::apra*	pLysS × AU1115 to Cm^r^
**MG1655 and derivatives**		
MG1655	*F^−^ rph-1 rec^+^ ruv^+^ rnhA^+^ polA^+^ lac^+^*	(42)
AM1780	*ΔpolA2::dhfr*	(27)
AM1783	*ΔlacIZYA ΔpolA2::dhfr*	P1.AM1780 × TB28 to Tm^r^
AM1955	*ΔruvABC::apra*	(37)
AM1968	pAM475 (*lac^+^ polA^+^*) / *ΔlacIZYA ΔpolA2::dhfr*	pAM475 × AM1783 to Ap^r^
AM1969	*Δrep::dhfr*	This work
AU1119	*ΔrecG263::kan* pDIM071	pDIM071 × N4256 to Ap^r^
AU1020	pJJ100 (*lac^+^ recG^+^*) / *ΔlacIZYA ΔrnhA::cat*	P1.N4704 × N6283 to Cm^r^ Ap^r^
AU1120	pDIM113 (eYFP-RecG eCFP-SeqA) / *ΔrecG263::kan*	pDIM113 × N4256 to Ap^r^
AU1122	pDIM133 (eYFP-RecG[Δwedge]) / *ΔrecG263::kan*	pDIM133 × N4256 to Ap^r^
AU1158	pAU110 (eYFP-RecG[ΔC5]) / *ΔrecG263::kan*	pAU110 × N4256 to Ap^r^
AU1159	pAU111 (eYFP-RecG[ΔC10]) / *ΔrecG263::kan*	pAU111 × N4256 to Ap^r^
AU1160	pAU112 (eYFP-RecG[ΔC15]) / *ΔrecG263::kan*	pAU112 × N4256 to Ap^r^
AU1194	*recG[ΔC5]-kan*	This work
AU1196	*recG[ΔC15]-kan*	This work
AU1200	*recG[ΔC5]-kan*	P1.AU1194 × MG1655 to Km^r^
AU1202	*recG[ΔC15]-kan*	P1.AU1196 × MG1655 to Km^r^
AU1210	pJJ100 (*lac^+^ recG^+^*) / *ΔlacIZYA ΔrnhA::cat recG[ΔC5]-kan*	P1.AU1194 × AU1020 to Km^r^
AU1216	*recG^+^-kan*	This work
AU1217	pJJ100 (*lac^+^ recG^+^*) / *ΔlacIZYA ΔrnhA::cat recG^+^-kan*	P1.AU1216 × AU1020 to Km^r^
AU1218	*recG^+^-kan*	P1.AU1216 × MG1655 to Km^r^
AU1219	*recG[ΔC5]-kan ΔruvABC::apra*	P1.AM1955 × AU1200 to Apra^r^
AU1221	*recG[ΔC15]-kan ΔruvABC::apra*	P1.AM1955 × AU1202 to Apra^r^
AU1232	*recG^+^-kan ΔruvABC::apra*	P1.AM1955 × AU1218 to Apra^r^
AU1238	pAM475 (*lac^+^ polA^+^*) / *ΔlacIZYA ΔpolA2::dhfr recG^+^-kan*	P1.AU1216 × AM1968 to Km^r^
AU1239	pAM475 (*lac^+^ polA^+^*) / *ΔlacIZYA ΔpolA2::dhfr recG[ΔC5]-kan*	P1.AU1194 × AM1968 to Km^r^
AU1241	*recG[Y690A]-kan*	This work
AU1244	*recG[Y690A]-kan*	P1.AU1241 × MG1655 to Km^r^
AU1247	*ΔruvABC::apra recG[Y690A]-kan*	P1.AU1241 × AM1955 to Km^r^
AU1250	pJJ100 (*lac^+^ recG^+^*) / *ΔlacIZYA ΔrnhA::cat recG[Y690A]-kan*	P1.AU1241 × AU1020 to Km^r^
JIG874	*recG[ΔC1]-kan*	This work
JIG876	*recG[ΔC1]-kan*	P1.JIG874 × MG1655 to Km^r^
JIG878	*ΔruvABC::apra recG[ΔC1]-kan*	P1.JIG874 × AM1955 to Km^r^
JIG880	pJJ100 (*lac^+^ recG^+^*) / *ΔlacIZYA ΔrnhA::cat recG[ΔC1]-kan*	P1.JIG874 × AU1020 to Km^r^
JIG893	*recG[A693Q]-kan*	This work
JIG895	*recG[A693Q]-kan*	P1.JIG893 × MG1655 to Km^r^
JIG896	*ΔruvABC::apra recG[A693Q]-kan*	P1.JIG893 × AM1955 to Km^r^
JIG897	pJJ100 (*lac^+^ recG^+^*) / *ΔlacIZYA ΔrnhA::cat recG[A693Q]-kan*	P1.JIG893 × AU1020 to Km^r^
JIG898	*recG[R682A,W683S]-kan*	This work
JIG899	*recG[R682A,W683S]-kan*	P1.JIG898 × MG1655 to Km^r^
JIG900	*ΔruvABC::apra recG[R682A,W683S]-kan*	P1.JIG898 × AM1955 to Km^r^
JIG901	pJJ100 (*lac^+^ recG^+^*) / *ΔlacIZYA ΔrnhA::cat recG[R682A,W683S]-kan*	P1.JIG898 × AU1020 to Km^r^
JIG911	pAM475 (*lac^+^ polA^+^*) / *ΔlacIZYA ΔpolA2::dhfr recG[Y690A]-kan*	P1.AU1241 × AM1968 to Km^r^
JIG915	pAM475 (*lac^+^ polA^+^*) / *ΔlacIZYA ΔpolA2::dhfr recG[ΔC1]-kan*	P1.JIG874 × AM1968 to Km^r^
JIG917	pAM475 (*lac^+^ polA^+^*) / *ΔlacIZYA ΔpolA2::dhfr recG[A693Q]-kan*	P1.JIG893 × AM1968 to Km^r^
JIG918	pAM475 (*lac^+^ polA^+^*) / *ΔlacIZYA ΔpolA2::dhfr recG[R682A,W683S]-kan*	P1.JIG898 × AM1968 to Km^r^
JIG920	pJG084 (eYFP-RecG[R682A,W683S]) / *ΔrecG263::kan*	pJG084 × N4256 to Ap^r^
JIG979	pJG137 (eYFP-RecG[R682L]) / *ΔrecG263::kan*	pJG137 × N4256 to Ap^r^
JIG980	pJG138 (eYFP-RecG[W683S]) / *ΔrecG263::kan*	pJG138 × N4256 to Ap^r^
JIG1021	*eYFP-recG-kan*^c^	This study
JIG1047	pJG155 (eYFP-RecG[W683L]) / *ΔrecG263::kan*	pJG155 × N4256 to Ap^r^
JIG1048	pJG156 (eYFP-RecG[W683F]) / *ΔrecG263::kan*	pJG156 × N4256 to Ap^r^
N4256	*ΔrecG263::kan*	(26)
N4583	*ΔruvABC::cat*	(57)
N4704	*ΔrnhA::cat*	(37)
N4971	*ΔrecG263::kan ΔruvABC::cat*	(57)
N6052	*ΔrecG::apra*	(27)
N6283	pJJ100 (*lac^+^ recG^+^*) / *ΔlacIZYA*	(37)
N7991	*ΔlacIZYA recG[ΔC1]-kan*	P1.JIG874 × TB28 to Km^r^
N7992	pJJ100 (*lac^+^ recG^+^*) / *ΔlacIZYA recG[ΔC1]-kan*	pJJ100 × N7991 to Ap^r^
N7993	pJJ100 (*lac^+^ recG^+^*) / *ΔlacIZYA recG[ΔC1]-kan ΔpolA2::dhfr*	P1.AM1780 × N7992 to Tm^r^
N8005	*ΔlacIZYA recG[ΔC1]-kan ΔpolA2::dhfr*	Plasmid free derivative of N7993^b^
N8202	*ΔlacIZYA ΔpolA2::dhfr recG^+^-kan*	Plasmid free derivative of AU1238^b^
N8203	*ΔlacIZYA ΔpolA2::dhfr recG[R682A,W683S]-kan*	Plasmid free derivative of JIG918^b^
RCe371	*Δrep::dhfr* pDIM113	pDIM113 × AM1969 to Ap^r^
RCe452	*ΔrecG::apra*	MG1655 × P1.N6052 to Apra^r^
RCe571	*Δrep::dhfr*	MG1655 × P1.RCe371 to Tm^r^
RCe575	*Δrep::dhfr* pDIM071	pDIM071 × RCe571 to Ap^r^
TB28	*ΔlacIZYA*	(40)

^a^The abbreviations *apra, cat, dhfr* and *kan* refer to insertions conferring resistance to apramycin (Apra^r^), chloramphenicol (Cm^r)^), trimethoprim (Tm^r^) and kanamycin (Km^r^), respectively. Ap^r^ refers to ampicillin resistance.

^b^From white colonies on minimal agar supplemented with X-gal and IPTG.

^c^The construct was generated by fusing the open reading frame for expressing eYFP to the 5′ end of a wild-type *recG* gene linked to a downstream *kan* sequence (see the Materials and Methods section) to give pJG146. The entire fusion linked to *kan* was amplified from pJG146 by PCR and directed by recombineering into N6052, selecting Km^r^ and screening for sensitivity to apramycin.

Plasmid constructs expressing fluorescent RecG fusions under control of the arabinose-inducible P*_araBAD_* promoter are derivatives of pLau18 (45). A sequence encoding the full length *recG* gene plus a 5′ extension encoding an N-terminal linker sequence (MELYLIDYLEC) was PCR amplified using a 5′ primer that also introduced a *Bsr*GI restriction site and a 3′ primer that introduced an *Xba*I site. The PCR product was cloned between the *Bsr*GI and *Xba*I sites of pLau18 such that the *recG* gene with its 5′ linker extension is inserted in frame downstream of the P*_araBAD_* promoter and eYFP coding sequence, generating pDIM071. To create constructs encoding the eYFP-RecG mutant fusions described in the main text, *Kpn*I-*Hin*dIII fragments from the pT7-7 constructs encoding the relevant mutant *recG* genes were cloned between the *Kpn*I and *Hin*dIII sites of pDIM071. pDIM083 (eCFP-SeqA) has been described previously (38). The *Nhe*I-*Hin*dIII fragment from pDIM083 was cloned between the *Xba*I and *Hin*dIII sites of pDIM071, generating pDIM113 (eYFP-RecG eCFP-SeqA).

### Media and general methods

LB broth and 56/2 minimal salts media and methods for monitoring cell growth and for strain construction by P1*vir*-mediated transduction have been cited (17,24,46). For microscopy, 56/2 salts were solidified with agarose at a final concentration of 1%.

### Fluorescence microscopy

Cultures of strains carrying the relevant fusion constructs were incubated in LB broth until they reached an A_650_ of 0.2. The encoded fusion proteins were induced by adding arabinose to a final concentration of 0.2% before incubating for a further 60 min. Samples were then transferred to a microscopic slide coated with a thin layer of 56/2 agarose. The cells were visualised using a BX-52 Olympus microscope equipped with a coolSNAP^TM^HQ camera (Photometrics). eCFP and eYFP foci were visualised using the JP4-CFP-YFP filter set 86002v2 (Chroma). Images were taken and analysed by MetaMorph 6.2 (Universal Imaging) and processed using MetaMorph and Adobe Photoshop.

### Measuring sensitivity to DNA damage

Sensitivity to UV light was measured using cultures of cells grown in LB broth to an A_650_ of 0.48 (1–2 × 10^8^ cells/ml for strain MG1655). Samples of appropriate dilutions were irradiated on the surface of LB agar plates and survivors were scored after 18–24 h incubation. Survival values are means of three to six independent experiments. Error estimates (SE) range from 5% to 15% of the mean. To determine sensitivity to mitomycin C, the cultures were diluted in 10-fold steps from 10^−1^ to 10^−5^, and 10 μl aliquots of each dilution spotted on LB agar with and without mitomycin C at a final concentration of 0.2 or 0.5 μg/ml, as indicated. Plates were photographed after 24 h incubation unless stated otherwise.

### Synthetic lethality assays

The assays were conducted as described (40,41). Essentially, a wild-type gene of interest is cloned in pRC7, an unstable *lac^+^* plasmid that is easily lost from cells, which is then used to cover a mutation of the same gene in the chromosome in a *Δlac* background. Plasmid loss is readily scored by spreading samples on agar plates supplemented with X-gal and IPTG. Plasmid-free cells form white colonies whereas cells retaining the plasmid form blue colonies with white sectors as plasmid loss continues during colony growth. If a mutation in another gene is introduced and the double mutant is viable, white colonies/sectors of plasmid-free cells still appear. However, if synthetically lethal, the plasmid-free cells fail to grow and only non-sectored blue colonies formed by cells retaining the plasmid are observed. The size of any Lac^−^ colonies relative to the Lac^+^ colonies also gives some indication of the viability of the plasmid-free cells. In our standard assays, cultures of strains carrying the relevant pRC7 derivatives were grown overnight in LB broth containing ampicillin to maintain plasmid selection, diluted 80-fold in fresh broth without ampicillin and incubation continued to an A_650_ of 0.48 before spreading samples of various dilutions on LB agar or 56/2 glucose minimal salts agar supplemented with X-gal and IPTG. Plates were photographed and scored after 48 h (LB agar) or 72 h (minimal agar) at 37°C. Plasmid-free cells forming small white colonies were re-streaked to see if they could be subcultured, and the streaked plates were photographed after incubation at 37°C for 24–48 h (LB agar).

### Purification of RecG

All chromatography was performed at 4°C and has been described previously (21). RecG proteins were expressed by IPTG induction from pT7-7 constructs in strain AU1118. Induced cells were resuspended in TNE (50 mM Tris-HCl pH 7.5, 100 mM sodium chloride, 1 mM EDTA), lysed by sonication and the supernatant was recovered by centrifugation (16 000 rpm, 4°C, 30 min) and filtered through a 0.45 μm syringe-end filter. The supernatant was loaded onto a 10 ml HiTrap SP HP column and eluted with a gradient of sodium chloride (0–1 M) in buffer A (50 mM Tris-HCl pH 7.5, 1 mM EDTA, 1 mM DTT). Fractions containing RecG were diluted with buffer A to final sodium chloride concentration of less than 150 mM and loaded onto a 5 ml HiTrap Heparin HP column. RecG was eluted with a gradient of sodium chloride (0–1 M) in buffer A. Fractions containing RecG were pooled and ammonium sulphate was added to a final concentration of 0.5 M before loading onto a 5 ml HiTrap Phenyl-Sepharose HP column and RecG was eluted with a stepped gradient of ammonium sulphate (0.5–0 M) in buffer A. Eluted RecG was collected and concentrated on a 5 ml HiTrap Heparin HP column attached downstream of the Phenyl-Sepharose column and eluted from the Heparin column as above. The eluted RecG was loaded onto a 16/60 Sephacryl S-200 HR column and eluted in gel filtration buffer (20 mM Tris-HCl pH 7.5, 150 mM sodium chloride). RecG was concentrated on a 5/5 Mono-S HR (1 ml) column and eluted with a gradient of sodium chloride (0–1 M) in buffer A. The pure RecG protein was dialysed overnight against two changes of storage buffer (50 mM Tris-HCl pH 7.5, 1 mM EDTA, 1 mM DTT, 100 mM sodium chloride, 50% glycerol) and stored at −80°C. Protein concentrations were determined using the Bradford assay with BSA as the standard.

### DNA substrates

DNA substrates were made by annealing the oligonucleotides, one of which was labelled with ^32^P at its 5′ end, as described (6). DNA concentrations are in moles of the molecular structure. J12 is a Holliday junction structure with a homologous core of 12 bp flanked by 19–20 bp heterologous arms (6). The fork structure mimics a replication fork lacking a lagging strand (21).

### DNA binding and unwinding assays

DNA binding by RecG was measured using a band-shift assay (6). RecG and ^32^P-labelled DNA were mixed in binding buffer (50 mM Tris-HCl pH 8.0, 5 mM EDTA, 1 mM DTT, 100 mg/ml BSA and 6% glycerol) and incubated on ice for 20 min before loading onto a pre-chilled 4% native polyacrylamide gel in a low ionic strength buffer (6.7 mM Tris-HCl pH 8.0, 3.3 mM sodium acetate and 2 mM EDTA). Electrophoresis was at 160 V for 75 min. Gels were then dried and analysed by autoradiography (X-Omat UV Plus film, Kodak) and phosphorimaging (STORM scanner system and ImageQuant 5.2, Molecular Dynamics). DNA unwinding was assayed essentially as described (12). The rates of unwinding were measured using bulk reactions. RecG at 0.5 nM was mixed in helicase buffer (20 mM Tris-HCl pH7.5, 2 mM DTT, 100 mg/ml BSA, 5 mM ATP and 5 mM MgCl_2_) and kept on ice prior to addition of labelled substrate DNA (0.2 nM). An aliquot was removed immediately and deproteinized by the addition of 0.2 volume of stop buffer (2.5% SDS, 200 mM EDTA and 10 mg/ml proteinase K) and incubating for a further 10 min at 37°C; this was taken as the time zero sample. The reaction was then placed at 37°C and samples subsequently removed at intervals and deproteinized. Samples were analysed by electrophoresis using a 10% polyacrylamide gel and a Tris-borate buffer system before processing as above.

## RESULTS

### RecG is located to sites of active DNA replication

**Figure 1.**
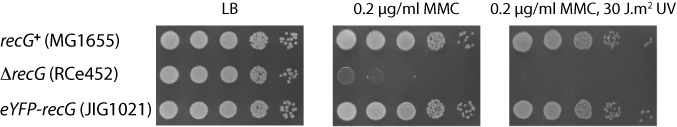
A chromosomal copy of *eYFP-recG* confers resistance to mitomycin C and UV light. The strains used are identified in parentheses.

We fused eYFP to the N-terminus of RecG and investigated whether the fusion protein forms foci that might reflect the role of RecG *in vivo*. A linker peptide was employed to construct the fusion protein because previous attempts to create direct fusions to the N-terminus of *E. coli* RecG failed, suggesting that such fusions might be lethal (47). The linker used is based on the peptide sequence linking the DNA binding domain of *Thermotoga maritima* RecG to an N-terminal domain that does not exist in the *E. coli* protein (22). We reasoned that the linker might keep the eYFP from interfering with RecG function and generally out of harm's way. We replaced the chromosomal copy of *recG* with our fusion construct. Strains expressing the fusion protein from the native *recG* promoter proved as resistant to mitomycin C and to UV irradiation as a strain carrying wild-type *recG*, showing none of the sensitivity of a *ΔrecG* strain and confirming that the eYFP-RecG fusion protein is indeed functional *in vivo* (Figure 1). However, we were unable to detect any foci by fluorescence microscopy (data not shown).

**Figure 2.**
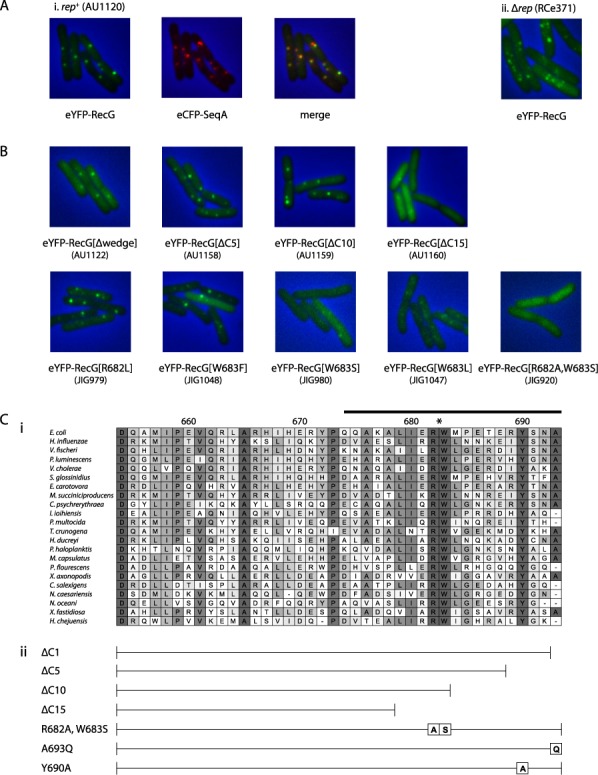
Cellular localisation of RecG. (**A**) RecG co-localises with SeqA. The strains identified in parentheses carry a construct expressing both eYFP-RecG and eCFP-SeqA. The panels show phase contrast images merged with the indicated fluorescence images. (**B**) Co-localisation depends upon the C-terminus of RecG. The panels show merged phase contrast and fluorescence images, with the constructs identified underneath. (**C**) Identification and mutation of conserved residues within C-terminus of RecG. (i) Multiple alignment of C-terminal sequences of RecG proteins. The sequences are from RecG proteins that have a C-terminal region of similar length. Shading is related directly to amino acid similarity. Residues are numbered according to *E. coli* RecG. The structure associated with residues in the region corresponding to the last 20 amino acids (marked by a black line above the *E. coli* sequence) has not been resolved at the atomic level (22). (ii) Schematic representation of the major C-terminal RecG deletions and substitutions used.

RecG is present at very low levels (less than 10 molecules per cell) (48), which might explain the absence of a fluorescence signal. Indeed, a recent study in which a more sensitive fluorescence microscopy system was used also revealed that a RecG fusion protein expressed from its native promoter does not form any detectable foci (49). Therefore, we over-expressed the fusion protein from a multi-copy plasmid using the P*_araBAD_* promoter. Its expression eliminated the sensitivity of a *ΔrecG* strain to UV irradiation and mitomycin C (data not shown). It also resulted in the formation of clearly defined foci in the majority of the cells analysed, anything from 1 to 6 per cell (Figure 2A(i)). A sample of 382 cells scored from three independent experiments revealed that 65% had 1 or 2 foci, 14.7% had 3 or 4 and 1.3% had 5 or 6. The remaining 19% had none. We next examined whether RecG foci co-localise with foci formed by SeqA protein tagged with eCFP (Figure 2A(i)). Of 323 RecG foci analysed in 164 cells from three independent experiments, 296 co-localised with SeqA foci. SeqA binds to hemi-methylated DNA, as found immediately behind replication forks (50,51), and therefore provides a marker for areas of the nucleoid in which replication is taking place. To gain further evidence that the location of RecG foci corresponds to sites of replication, we exploited the fact that chromosome copy number is increased in cells lacking the DNA helicase Rep (52). If RecG does accumulate at sites of replication, there should be an increase in foci following expression of the fusion protein in these cells. This is what we observed (Figure 2A(ii)). An analysis of 186 cells sampled from two independent experiments with a *Δrep* strain revealed that only 38.5% of the cells had 1 or 2 foci. Sixty percent had 3 or more, with 12% showing 5 or 6. Only 1.5% of the cells had no foci at all, which is less than a tenth of the number seen with *rep^+^* cells.

Both the cellular location and number of these RecG foci are consistent with the idea that the protein associates with replisome complexes, as RecG is reported to do in *B. subtilis*. Lecointe *et al.* (53) demonstrated that *B. subtilis* RecG co-localises with the replisome protein DnaX via an interaction with the C-terminus of the single-stranded DNA binding protein SSB (53). Recent studies have shown that *E. coli* RecG also interacts with the C-terminus of *E. coli* SSB *in vitro* (27,54–55). Given that SSB is concentrated at sites of active replication (56), this interaction might account for the RecG foci we observed, and for their co-localisation with SeqA. We can exclude localisation via a protein:DNA interaction as we found that an eYFP-RecG construct based on a RecG derivative lacking the ‘wedge domain’ needed for high affinity DNA binding still localises into foci (48) (Figure 2B).

Co-localisation of RecG with the replisome via an interaction with SSB might explain the previously observed lethal effect of a direct fusion of maltose binding protein (MBP) to the N-terminus of RecG (47). The fusion could be lethal because the MBP tag might disturb vital replisome interactions. Since the eYFP-RecG described here was expressed without causing loss of viability, we assumed the extra linker region deployed between the two globular proteins might be holding the eYFP tag in a position where it does little or no harm. Significantly, the failure to create a direct fusion of MBP to RecG was overcome by deleting the last 32 amino acid residues from the C-terminus of RecG. The MBP-RecGΔC32 mutant protein still bound branched DNA *in vitro* with an affinity similar to that of wild-type RecG (47). However, localisation of the construct *in vivo* was never investigated. If some feature of the C-terminus of RecG mediates the interaction with SSB, mutant RecG proteins with deletions or substitutions eliminating this feature would no longer be expected to exhibit localisation.

### C-terminal mutations reduce the ability of RecG to form foci

We generated a set of *recG* alleles encoding eYFP-RecG fusions lacking the last 5, 10, 15, 20 or 30 amino acids (Figure 2C(ii)). Proteins lacking the last 5 or 10 residues still form discrete foci, but a protein lacking the last 15 was mostly distributed throughout the cell, with foci barely visible (Figure 2B). More extensive deletions eliminated focus formation altogether (data not shown). An alignment of the C-terminus of RecG proteins that have C-termini of similar length shows that a tryptophan (W) and an arginine (R), located 11 and 12 residues respectively from the C-terminus of *E. coli* RecG, are conserved (Figure 2C(i)). We created mutant proteins with substitutions at one or both of these positions and examined eYFP derivatives to see if they would still localise. Focus formation was still evident with R682L and W683F proteins, but less so with W683L and W683S derivatives, and hardly detectable with a protein carrying both R682A and W683S substitutions (Figure 2B). This double mutant, which we designate as RecG[RW], was clearly distributed throughout the cell.

These data indicate that arginine 682 and tryptophan 683 might indeed be key to focus formation. They are consistent with the notion that the foci we observe with eYFP fused to wild-type RecG reflect localisation of RecG to specific sites rather than being caused by aggregation of the fluorescent fusion. Such localisation might be facilitated via an interaction between the C-terminus of RecG and that of SSB. The fact that the arginine and the tryptophan are conserved in RecG proteins from diverse species indicates that localisation of RecG might be generally important.

### Effect of localisation on the *in vivo* activity of RecG

**Figure 3.**
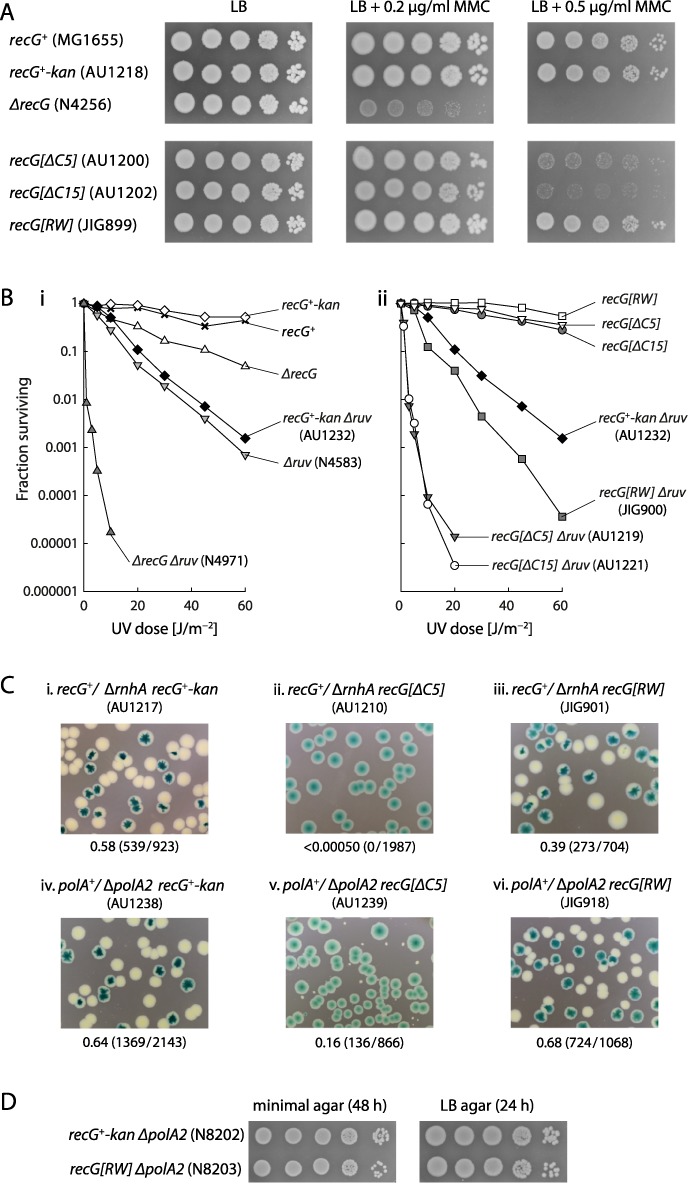
Effect of RecG C5 and C15 deletions and RW substitutions on sensitivity to DNA damage and viability. (**A**) Sensitivity to mitomycin C. (**B**) Sensitivity to UV light. The strains used were as in (A) plus additional constructs identified in parentheses. Data for strain AU1232 are duplicated in panels (i) and (ii) for the purposes of comparison. (**C**) Synthetic lethality assays illustrating the effect of the C-terminal RecG mutations on the viability of *ΔrnhA* and *ΔpolA2* strains on LB agar. The relevant genotype is shown above each photograph, with the strain number shown in parentheses. The fraction of white colonies is shown below, with the number of white colonies/total colonies analysed shown in parentheses. (**D**) Relative plating efficiencies of *ΔpolA2 recG^+^-kan* and *ΔpolA2 recG[RW]* cells. Cultures of the strains identified were grown in 56/2 glucose minimal salts medium to an A_650_ of 0.48, diluted in 10-fold steps from 10^−1^ to 10^−5^, and 10 μl aliquots spotted on minimal and LB agar, as indicated. Plates were photographed after incubation for 24 h (LB agar) or 48 h (minimal agar).

To assess whether localisation has any effect on RecG function, we analysed the phenotype conferred by the C-terminal mutations. Each mutation was engineered at the chromosomal *recG* locus under its native promoter, linked to a downstream *kan* insertion. A wild-type *recG* sequence was similarly linked to *kan* to provide an appropriate control, and is designated *recG^+^-kan*. Replacing *recG^+^* with *recG^+^-kan* does not increase sensitivity to mitomycin C (Figure 3A), a major feature of *recG* null strains (1). Furthermore, it does not increase the sensitivity of a *ΔruvABC* strain to UV light (Figure 3B(i)), a particularly sensitive test of RecG activity given the very strong synergism between *recG* and *ruv* null alleles (5,57). Thus, it seems the *kan* insertion engineered downstream of *recG* has little or no effect on the activity of the gene.

Strains carrying *ΔC20* and *ΔC30* alleles of *recG* proved sensitive to mitomycin C and UV light, and each allele conferred extreme UV sensitivity when combined with Δ*ruvABC* (data not shown), consistent with these alleles retaining little or no RecG activity. However, we cannot attribute this to the failure of localisation as previous studies suggested that substantial deletions from the C-terminus severely curtail *in vitro* DNA unwinding activity (43). We therefore investigated the phenotype of an R682A, W683S double mutant, which greatly reduces the ability to form foci. Despite this failure to localise, the RecG[RW] protein appears to retain much of its ability to function. Thus, a strain carrying the mutant allele (*recG[RW]*) grows robustly on agar plates supplemented with mitomycin C (Figure 3A). On its own, the *recG[RW]* allele confers no sensitivity to UV light and although it does increase sensitivity when combined with *ΔruvABC*, it does so only to a slight extent (Figure 3B(ii)). To gain further measures of activity, we exploited the fact that a *recG* null allele confers lethality when combined with *rnhA* or *polA* deletions (27,37,58). An assay for synthetic lethality based on unstable pRC7 derivatives carrying either *recG^+^* or *polA^+^* revealed that *recG[RW] ΔrnhA* and *recG[RW] polA2* double mutant cells are quite viable, establishing robust colonies without a covering plasmid (Figure 3C, panels (iii) and (vi)), colonies that can be subcultured without difficulty (Figure 3D). Thus, RecG[RW] does not appear severely compromised by the reduced ability to form foci.

Genetic analyses of strains carrying *recG[ΔC5]* or *recG[ΔC15]* revealed that while they appear to differ in their ability to form foci, they confer equally strong mutant phenotypes, much stronger than is conferred by *recG[RW]*. Both confer more resistance to mitomycin C and UV light than does a *recG* null allele (*ΔrecG*) (Figure 3A and B). However, when combined with *ΔruvABC*, they both confer extreme sensitivity to UV light, almost as extreme as that of a *ΔrecG ΔruvABC* strain (Figure 3B). Unlike *recG[RW]*, both alleles also proved synthetically lethal with *ΔrnhA* and also with *ΔpolA2* (Figure 3C and data not shown). The small white colonies seen in the *ΔpolA2* assay (Figure 3C(v)) could not be subcultured.

Taken together, these data indicate that there is no strong correlation between RecG focus formation and RecG activity. However, the slightly increased sensitivity to UV light of the *recG[RW] ruvABC* double mutant means we cannot dismiss entirely the possibility that the localisation of RecG confers some advantage.

### The extreme C-terminus of RecG is necessary for function

**Figure 4.**
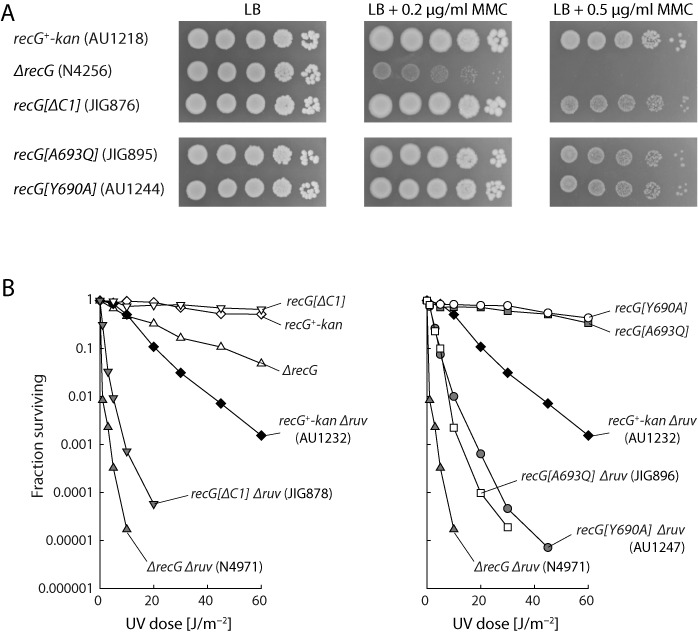
Effect of extreme RecG C-terminal mutations on sensitivity to DNA damage. (**A**) Sensitivity to mitomycin C. The strains used are identified in parentheses. (**B**) Sensitivity to UV light. The strains used were as in (A) plus additional constructs identified in parentheses. Data for strains AU1218, AU1232, N4256 and N4971 are reproduced from Figure 3B for the purposes of comparison.

**Figure 5.**
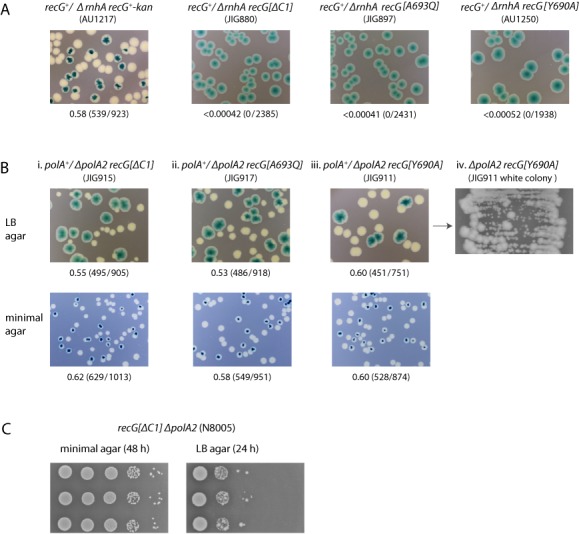
Effect of extreme RecG C-terminal mutations on the viability of *ΔrnhA* and *ΔpolA2* cells. (**A**) and (**B**) Synthetic lethality assays. The relevant genotype is shown above each photograph, with the strain number shown in parentheses. The fraction of white colonies is shown below, with the number of white colonies/total colonies analysed shown in parentheses. (A) Assays with *ΔrnhA* constructs on LB indicator plates. The data for strain AU1217 are reproduced from Figure 3C(i) for comparison. (B) Assays with *ΔpolA2* constructs on both LB (top row) and 56/2 glucose minimal salts indicator plates. Panel (iv) illustrates the formation of large colonies by faster growing variants (suppressors) accumulating in the white colonies shown in panel (iii). (**C**) Low viability of *recG[ΔC1] ΔpolA2* cells on LB agar. Three independent colonies of plasmid-free *recG[ΔC1] ΔpolA2* cells established on 56/2 glucose minimal salts indicator plates were inoculated into 56/2 glucose minimal salts and incubated until the cell density reached an A_650_ of 0.48. Samples were then diluted in 56/2 salts in 10-fold steps from 10^−1^ to 10^−5^, before spotting 10 μl aliquots of each dilution on 56/2 glucose minimal salts agar and LB agar, as indicated. These plates were photographed after 24 and 48 h incubation, respectively.

The strong phenotype conferred by *recG[ΔC5]* prompted us to further dissect the C-terminus. We first examined the effect of adding three alanine residues to the very end. Tests for sensitivity to mitomycin C and UV light, and for viability in the presence of *ΔrnhA* or *ΔpolA2*, revealed no substantial reduction in RecG functionality *in vivo* (data not shown). However, removing one, two or three residues from the C-terminus clearly reduced the ability of the protein to function. The phenotype conferred in each case proved essentially identical and so we restrict our data presentation to *recG[ΔC1]*, which removes the terminal alanine (Figure 2C). We also report on two missense alleles, one substituting the conserved tyrosine four residues from the end with an alanine (*recG[Y690A]*) and the other substituting the terminal alanine with a glutamine (*recG[A693Q]*). Single mutants carrying any one of these three alleles proved only very mildly sensitive to mitomycin C and quite resistant to UV (Figure 4A and B). But a substantially reduced RecG activity was uncovered when these alleles were combined with *ΔruvABC*. In each case, the double mutant proved very much more sensitive to UV light than a *ΔruvABC* control (Figure 4B). Synthetic lethality assays also revealed a loss of functionality. The assays showed that strains carrying these alleles require wild-type *rnhA* to grow on LB agar (Figure 5A). Similar assays testing viability with *ΔpolA2* proved deceptive. In this case, the double mutant cells form what appear to be reasonably robust (white) colonies on the LB indicator plates (Figure 5B(i)–(iii)), giving the impression that DNA polymerase I is dispensable. However, when these colonies are streaked on LB agar, it becomes apparent that much of the colony growth can be attributed to abortive growth of inviable cells and the outgrowth of suppressors (Figure 5B(iv)). Further inspection revealed that the double mutants are viable on minimal agar and can be subcultured without being outgrown by suppressors, as evident when these subcultures are subsequently tested for colony formation (the same is true for *ΔrecG ΔpolA2* cells). The vast majority of the cells fail to establish colonies on LB agar, but grow fine on minimal agar (Figure 5C), demonstrating that DNA polymerase I is in fact needed to sustain viability under conditions supporting rapid growth and division.

### RecG C-terminal mutations compromise biochemical activity

Previous studies established that the helicase domains of RecG are located towards the C-terminus, with the N-terminal wedge domain being involved with DNA binding (20–22,43,48). Therefore, we suspected that the substantial *in vivo* reduction in RecG activity observed with each of the three C-terminal mutants described in the previous section is due to a reduction in the ability to unwind DNA. Unfortunately, the extreme C-terminus of RecG is not resolved in the only published crystal structure of RecG (22), indicating a degree of flexibility. Therefore, its precise disposition relative to the conserved helicase domains is not known.

**Figure 6.**
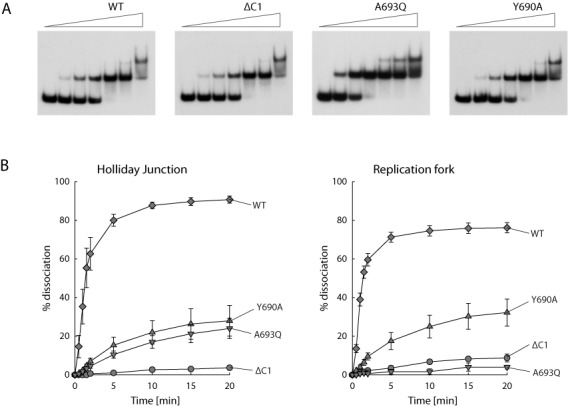
DNA binding and unwinding activities of RecG C-terminal mutants. (**A**) DNA binding assays. The autoradiograph shown is of representative band-shift assays with Holliday junction substrate J12. Each set of reactions from left to right used the RecG proteins indicated at 0, 0.1, 0.4, 1.6, 6.4, 25 and 100 nM and ^32^P-labelled J12 DNA at 0.2 nM. (**B**) DNA unwinding assays. The graphs show the rates of dissociation of ^32^P-labelled Holliday junction and replication fork substrates by the proteins indicated. Reactions contained substrate DNA at 0.2 nM and RecG protein at 0.5 nM. Data are means (±SE) of at least three independent experiments.

We purified some of the mutant RecG proteins and investigated their ability to bind and unwind branched DNA substrates. The ΔC1, Y690A and A693Q proteins retain the ability to bind the Holliday junction and fork DNA structures tested, and with an affinity indistinguishable from wild-type RecG (Figure 6A and data not shown). They also retain some unwinding activity, but this is substantially reduced (Figure 6B). RecG[ΔC1] has the most extreme deficiency in that it unwinds very little of either of the two substrates. RecG[A693Q] also shows little unwinding of the fork, but retains some activity on the Holliday junction. RecG[Y690A] has an intermediate activity with both substrates. Taken together with the *in vivo* properties, these data demonstrate that the extreme C-terminus of RecG down to the very last amino acid is necessary for the protein to be fully functional. Given this fact, we were surprised that adding three alanine residues to the C-terminus has no negative effect that we could detect. As with the *in vivo* studies, the extended protein is as active as wild-type RecG in the binding and unwinding assays (data not shown).

## DISCUSSION

### Localisation of *E. coli* RecG

Several lines of evidence have led to the suggestion that RecG might have a particularly important role to play in securing faithful genome duplication (2,39). The observation that *Bacillus subtilis* RecG co-localises with the replisome lends support to this idea (53), suggesting that the function of RecG is required specifically where DNA replication is taking place.

The data presented in this study support the idea that *E. coli* RecG also localises to the replisome (Figure 2). We found that an eYFP-RecG fusion forms foci that co-localise with SeqA, a marker for sites of active DNA synthesis. We were concerned initially that the foci observed might be the result of fluorescent protein dimerization and subsequent aggregation due to over-expression (59). However, the correlation with chromosome copy number revealed using a strain lacking Rep helicase and the elimination of foci by specific C-terminal mutations indicates that they are instead the result of a specific localisation to sites of chromosome replication.

Two conserved and adjacent amino acid residues, Arginine 682 and Tryptophan 683, were identified as being particularly important effectors of this localisation. This is significant as these residues lie close to the very end of the protein in a region that is not resolved in the RecG crystals described to date (22). Therefore, although the structure and disposition of this region has not been established, it is most likely flexible and free to interact with other factors. Given *E. coli* RecG interacts with SSB protein *in vitro* (27,54), and SSB coats the unwound lagging strand at forks, it is tempting to speculate that the observed localisation of RecG reflects this interaction, with the two proteins making contact via their C-termini.

However, our analyses of C-terminal RecG mutants provided no evidence of a strong correlation between localisation and function. Our C-terminal deletion mutants were not particularly informative in this respect as all those constructed severely curtail the activity of the protein *in vivo*. With hindsight, this is perhaps not surprising as we found that deleting just the very last amino acid is sufficient to compromise activity both *in vivo* and *in vitro*, as we discuss below. What we did find is that the RecG[RW] substitution mutant localises very poorly compared with the wild-type protein despite otherwise retaining near wild-type activity *in vivo*. The properties associated with this protein question the significance of the observed localisation. Does it really signify a role for RecG in promoting rescue of stalled forks as suggested (17,25,60)? The fact that RecG interacts with SSB allows for alternative interpretations.

During balanced growth, SSB is particularly concentrated at replication forks where it associates with the unwound lagging strand template (56,61). The observed localisation of the over-expressed *E. coli* RecG to the replisome may simply reflect this fact. Given the very high affinity of SSB for ssDNA and assuming it does act as a hub for DNA repair proteins *in vivo*, as suggested (55), there is no reason why the bound RecG should not re-locate to wherever ssDNA is exposed and covered with SSB. Thus, RecG could be targeted to any number of branched DNA substrates (2), only some of which may be the ‘true’ substrate at which RecG function is required. However, the conservation of the RW motif near the C-terminus of RecG, coupled with the slight loss of RecG function when this motif is mutated, indicates that there may be some selective advantage to the co-localisation with SSB. For instance, the SSB interaction might enhance RecG helicase activity, as recently reported (62).

### RecG helicase activity

We found that the extreme C-terminus of RecG is crucial for helicase activity *in vitro*. Previous analysis of deletion mutants had established the importance of the C-terminus, but the shortest deletion tested removed 32 residues from the end of the protein (43). Here we describe how eliminating just the terminal alanine is sufficient to make the protein almost non-functional *in vitro*, despite showing no reduction in substrate affinity (Figure 6). Substituting a glutamine for this alanine has almost as drastic an effect, especially with a fork substrate. This was rather surprising, as the respective single mutant strains exhibited only very mild sensitivity to mitomycin C and appeared fully resistant to UV light (Figure 4), which seemed to indicate that the proteins retained substantial activity. The presence of SSB, which has been reported to stimulate RecG activity (62), may account for this discrepancy. However, more sensitive assays based on double mutants carrying additional mutations inactivating RuvABC, ribonuclease HI or DNA polymerase I established that there is in fact a substantial loss of function *in vivo* (Figures 4 and 5). Nevertheless, we note that this alanine is not uniformly present in closely related RecG proteins (Figure 2C).

In conclusion, our data demonstrate that the C-terminus of RecG is vital for both its helicase activity and for its localisation. However, while a reduced helicase activity is clearly associated with a distinct mutant phenotype, there appears to be no strong correlation between localisation and functionality.
